# Editorial: Involvement of Autophagy in Determining the Developmental Fate of Ovarian Follicles

**DOI:** 10.3389/fcell.2021.814135

**Published:** 2021-12-23

**Authors:** Jia-Qing Zhang, Karin Lykke-Hartmann, Ming Shen

**Affiliations:** ^1^ Institute of Animal Husbandry and Veterinary Science, Henan Academy of Agricultural Sciences, Zhengzhou, China; ^2^ Department of Biomedicine, C Aarhus University, Aarhus, Denmark; ^3^ Department of Clinical Medicine, Aarhus University, Aarhus, Denmark; ^4^ Department of Clinical Genetics, Aarhus University Hospital, Aarhus, Denmark; ^5^ College of Animal Science and Technology, Nanjing Agricultural University, Nanjing, China

**Keywords:** follicular development, granulosa cell survival, oocyte aging, luteinization, hypoxia, autophagy, cell signalling

Autophagy is an evolutionary conserved catabolic mechanism in eukaryotes that involves recycling of cytoplasmic proteins and organelles to maintain cellular homeostasis. Over the past decade, studies have demonstrated that autophagy governs an array of physiological processes in follicular development, ovulation and luteinization. This special issue contains a collection of six original research papers and three abstracts covering a broad range of topics providing novel insights into the mechanisms of autophagy in regulating the physiological functions of ovarian follicular cells.

Qiang Liu et al. explored the molecular relationships among FSH, progesterone production, lipid droplet (LD) degradation, and autophagy in porcine granulosa cells (GCs) (Liu et al.). They found that FSH activated the PI3K/AKT and SAPK/JNK pathways to stimulate BECLIN1 expression, leading to upregulated autophagy and LD degradation, which promoted progesterone production. Although FSH treatment could also significantly increase the mRNA levels of StAR and P450scc, inhibiting autophagy using chloroquine or JNK antagonist in FSH-treated GCs abolished FSH-induced progesterone production, while the expression of StAR and P450scc was not disturbed. This result further suggested that autophagy might play a role in providing for the increased substrate requirements resulting from FSH-induced expression of steroidogenesis enzymes. Together, this study provides evidence demonstrating a novel role of autophagy in FSH-regulated steroid production.

Two articles by Tang et al. focused on the role of autophagy under hypoxic conditions in the rat ovary, firstly in the avascular granulosa layer of the developing follicle and secondly during the early formation of the corpus luteum (Tang et al.). The authors cultured the GCs from antral follicles and granulosa-lutein cells from developing corpora lutea under 3% oxygen or CoCl_2_ treatment to mimic hypoxia *in vitro*. The studies revealed that hypoxia induces in both *in vitro* culture systems HIF-1alpha/BNIP3 signaling resulting in elevation of autophagy. Therefore, autophagy appears to be essential for GC survival during folliculogenesis and for the luteinisation of GCs after ovulation.

Follicular atresia is the ultimate fate of most ovarian follicles (>99%) in mammalian ovary (Baker, 1963). Protein phosphorylation has been proven to be involved in regulating follicular atresia (Yang et al., 2020a). However, there have been very few studies aimed at characterizing the phosphoproteomic dynamics during follicular atresia. Utilizing TMT labeling, enrichment of phosphopeptides and LC-MS/MS analysis, Yang et al. conducted a proteomic/phosphoproteomic profiling of GCs from the healthy, slightly atretic, and atretic follicles in porcine ovaries. Several differentially expressed proteins (DEPs) and phosphorylated proteins (DEPPs) were identified as potential novel biomarkers to distinguish follicles of different atretic statuses. Based on mechanism research, they demonstrated that dephosphorylation of caspase six at Ser76 can increase the ratio of cleaved caspase6/caspase6 and lead to GCs apoptosis. Moreover, the phosphoproteomic data revealed some kinase regulatory networks of autophagic signaling. Compared with atretic follicles, the activation of PIK3C3 in healthy follicles was predicted to inhibit autophagy by catalyzing phosphorylation of autophagic regulators such as SQSTM1 (at S272) and AMBRA1 (at S52), indicating that autophagy might be involved in the atretic process. These findings might provide novel explanations regarding the regulation of follicular atresia.

It is still a matter of debate as to whether autophagy, not only facilitates corpus luteum formation by preventing apoptosis, but also promotes luteolysis through excessive cell destruction. The switch mechanism of “autophagic survival” and “autophagic death” during luteinization and luteolysis remains to be elucidated. Przygrodzka et al. explored the autophagic signaling pathways required for luteinization and luteolysis in steroidogenic cells of the corpus luteum. Their results indicated that luteotrophic signaling *via* a LH/PKA/MTOR pathway inhibits, while luteolytic signaling via a PGF2α/Ca2+/AMPK pathway activates key signaling pathways involved in luteal cell autophagosome formation. They demonstrated that autophagy-related signaling is upregulated during luteolysis, accompanied by cellular death. These findings extend the current knowledge on two prominent metabolic pathways, AMPK and PKA/MTOR signaling, in the maintenance or regression of the corpus luteum.

The autophagic activity has been found to decrease with age, likely leading to the accumulation of damaged macromolecules and organelles. Peters et al. investigated whether autophagy is involved in age-related oocyte quality decline, which is considered as a major contributor to female infertility. Characteristics of autophagy dysfunction, including decreased autophagosomes, decreased lysosomes, and amphisome accumulation, were detected in the aged oocytes. Interestingly, there are no significant differences in the expression of macroautophagy related proteins between young and aged mouse oocytes. However, a growing number of large autophagic puncta (>10 μm^2^) was observed in aged oocytes. Comprehensive image-based analysis revealed colocalization of biomarkers of ensosomes and autophagosomes, representing the formation of amphisomes. In addition, aged oocytes exhibit decreased numbers of lysomes, while lysosomal inhibition promotes the formation of large amphisomes. These findings provide a novel model linking the compromise of lysosomal function and the accumulation of amphisomes to the mechanism of oocyte aging.

Taken together, these research articles introduce readers to novel insights and important perspectives on the relationship between autophagy and ovarian physiology. We hope that this Research Topic will serve as a platform to initiate new research questions and innovative ideas in the field of female reproduction.
